# Mesenchymal stem cells activate Notch signaling to induce regulatory dendritic cells in LPS-induced acute lung injury

**DOI:** 10.1186/s12967-020-02410-z

**Published:** 2020-06-16

**Authors:** Zhonghua Lu, Shanshan Meng, Wei Chang, Shanwen Fan, Jianfeng Xie, Fengmei Guo, Yi Yang, Haibo Qiu, Ling Liu

**Affiliations:** Department of Critical Care Medicine, Zhongda Hospital, School of Medicine, Southeast University, 87 Dingjia Bridge, Hunan Road, Gu Lou District, Nanjing, 210009 China

**Keywords:** Acute respiratory distress syndrome (ARDS), Acute lung injury (ALI), Dendritic cells, Notch, Mesenchymal stem cell (MSC)

## Abstract

**Background:**

Mesenchymal stem cells (MSCs) have been shown to alleviate acute lung injury (ALI) and induce the production of regulatory dendritic cells (DCregs), but the potential link between these two cell types remains unclear. The goal of this study was to investigate the effect and mechanism of MSC-induced regulatory dendritic cells in ALI mice.

**Material/methods:**

In vivo experiments, C57BL/6 wild-type male mice were sacrificed at different times after intratracheal injection of LPS to observe changes in lung DC maturation and pathological damage. MSCs, DCregs or/and carboxyfluorescein diacetate succinimidyl ester (CFSE)-labeled DCs were administered to the mice by tail vein, and flow cytometry was performed to measure the phenotype of lung DCs and T cells. Lung injury was estimated by the lung wet weight/body weight ratio and histopathological analysis. In vitro, Western blotting or flow cytometry was used to detect the expression of Notch ligand or receptor in MSCs or DCs after coculture or LPS stimulation. Finally, in vivo and in vitro, we used the Notch signaling inhibitor DAPT to verify the effect of the Notch pathway on MSC-induced DCregs and their pulmonary protection.

**Results:**

We showed significant accumulation and maturation of lung DCs 2 h after intratracheal injection of LPS, which were positively correlated with the lung pathological injury score. MSC treatment alleviated ALI lung injury, accompanied by a decrease in the number and maturity of classical DCs in the lungs. CFSE-labeled DCs migrated to the lungs of ALI mice more than those of the normal group, and the elimination of CFSE-labeled DCs in the blood was slower. MSCs inhibited the migration of CFSE-labeled DCs to the lung and promoted their elimination in the blood. DCregs, which are obtained by contact coculture of mDCs with MSCs, expressed reduced levels of MHCII, CD86, CD40 and increased levels of PD-L1, and had a reduced ability to stimulate lymphocyte proliferation and activation (expression of CD44 and CD69). mDCs expressing Notch2 significantly increased after coculture with MSCs or rhJagged1, and MSCs expressed more Jagged1 after LPS stimulation. After stimulation of mDCs with recombinant Jagged1, DCs with low expression of MHCII, CD86 and CD40 were also induced, and the effects of both rhJagged1 and MSCs on DCs were blocked by the Notch inhibitor DAPT. Intra-airway DAPT reversed the inhibitory effect of mesenchymal stem cells on DC recruitment to the lungs and its maturation.

**Conclusions:**

Our results suggested that the recruitment and maturation of lung DCs is an important process in early ALI, MSCs attenuate LPS-induced ALI by inducing the production of DCregs by activating Notch signaling.

## Background

Acute respiratory distress syndrome (ARDS) is a common and clinically complex acute pulmonary inflammatory syndrome with a mortality rate of up to 40% [1]. Although after decades of exploration, there is still no special treatment for ARDS, treatment options are still confined to supportive care such as the protective mechanical ventilation and prone position ventilation and liquid conservative strategy [2]. Immune cell-mediated aberrant inflammatory responses play a significant role in the development of ARDS [3, 4], but glucocorticoid, surfactants, and other anti-inflammatory drugs, have been failed to show benefits [5]. In order to alleviate aberrant inflammatory of ARDS and ameliorate lung injury, it is urgent to explore additional therapeutic measures.

Mesenchymal stem cells (MSCS), with their pluripotent and immunomodulatory functions, are expected to be a novel therapy for ARDS. Our previous studies showed that MSC can repair alveolar epithelial cells and vascular endothelial cells to ameliorate lung injury in ARDS [6, 7]. MSCs also possess immunomodulatory properties to immune cells, such as T, B, NK cells, macrophages, neutrophils and dendritic cells (DCs) [8–11]. DCs are the most important specialized antigen presenting cells and play an important role in the regulation of lung immune response network. Previous studies have shown that the maturity of classical dendritic cells (cDCs) in the lungs of ALI mice increased significantly, while inhibition of DCs maturation reduced the inflammatory response and pathological damage in the lungs [12, 13]. MSC induced mature DCs (mDCs) differentiation into regulatory DC (DCreg) with low expression of CD80 and CD86 DCreg, which induced the expression of CD4, CD25, and Foxp3 in primary splenocytes isolated from mice [14]. MSC also abrogated the capacity of mDC migration to CCL19, or for DC to display MHC class II peptide complexes recognized by specific antibody, or ovalbumin-pulsed DC to support antigen-specific CD4^+^ T cell proliferation [15]. Therefore, transplantation of MSC to regulate the function of mature DC may bring new light for ARDS treatment, but the regulatory mechanism remains to be elucidated.

Many studies suggest that the immunosuppressive effect of MSCs occurs through paracrine or cell-to-cell contact mechanisms [16–18]. Cell–cell interactions have been demonstrated in the treatment of hematopoietic system-related diseases [16, 18]. The interaction between the Notch receptor and Notch ligand plays a key role in dendritic cell differentiation or MSC protection against inflammation [19, 20]. Cahill et al. [19] reported that MSCs prevented DC antigen presentation, and knocking down Jagged-1 in MSCs partially reversed this effect. The cell-binding Notch ligand activates notch-1 and notch-2 receptors, leading to accumulation of immature DCs [21]. Whether the Notch pathway is an important link in MSC-induced DCreg generation to alleviate acute lung injury remains unclear.

In the present study, we assessed changes in the lung cDC phenotype and their association with lung pathological injury in ALI mice, evaluated the effect of MSC transplantation on the lung cDC phenotype in ALI mice and the cellular mechanisms between them, and demonstrated that MSCs induce a shift in cDCs from a proinflammatory mature type to a tolerant DC to alleviate lung injury.

The aim of this study was to investigate whether the protective effect of MSCs on LPS-induced ALI was achieved by intercellular contact to induce mDCs to differentiate into DCregs and whether the mechanism was related to activation of the Notch pathway. We used coculture experiments to test the phenotype of dendritic cells, the stimulation of T cell proliferation, and the expression of PD-L1. To explore the mechanism of MSC-induced mDC conversion to DCregs to alleviate LPS-induced ALI, we used the Notch pathway inhibitor DAPT (a gamma secretase inhibitor) in vivo and in vitro and evaluated lung pathological injury, DC phenotype and function, and pulmonary edema. This study provides an immunological explanation for mesenchymal stem cell reduction of LPS-induced lung injury.

## Materials and methods

### Animals

Male wild-type 6- to 8-week-old BALB/c and C57BL/6 specific pathogen free (SPF) mice (Laboratory Animal Center of Yangzhou University, Yangzhou, China) were maintained under specific pathogen-free conditions. A SPF animal room and super-clean bench in the laboratory were used for animal feed and experimental manipulation respectively during the whole experiment. The Animal Care and Use Committee of Southeast University approved all of the experimental procedures (Ethics Committee Approval Number: 20170105007).

### MSC culture

Mouse mesenchymal stem cells and dendritic cells were used in the present study. MSCs derived from the bone marrow of C57BL/6 mice were purchased from Cyagen Biosciences Inc. (Guangzhou, China). The supplier identified MSCs based on the cell surface phenotype and pluripotency. MSCs were cultured in DMEM/F12 containing 10% fetal bovine serum (Wisent, Nanjing, China) and grown in a humidified 5% CO_2_ sterile incubator at 37 °C.

### Bone marrow isolation and dendritic cell culture

Bone marrow (BM)-derived DCs were generated as previously described with minor modifications [22, 23]. BM cells were extracted from the medullary cavity of the femur and tibia in super-clean bench. The erythrocytes were lysed using lysing buffer (BD Pharm Lyse™, USA), washed three times in phosphate-buffered saline (PBS) and cultured in 100 mm dishes with 2 × 10^6^ cells in fresh RPMI 1640 (Wisent, Nanjing, China), containing 10% FBS (Wisent, Nanjing, China), 40 ng/mL recombinant murine granulocyte–macrophage colony-stimulating factor (GM-CSF; NOVUS), and 40 ng/mL recombinant murine interleukin-4 (IL-4; NOVUS) in a humidified 5% CO_2_ sterile incubator at 37 °C. For the isolation of immature DCs (imDCs), non-adherent cells were gently washed away on day 3, half of the culture supernatant was collected and centrifuged, and the cell pellet was resuspended in 5 mL fresh medium as described above and returned to the original plate at day 5. The remaining loosely adherent cell clusters were collected and purified by anti-CD11c micromagnetic beads (Miltenyi Biotec) on day 6. Purified imDCs were cultured for an additional 24 h under stimulation with 50 ng/mL bacterial lipopolysaccharide (LPS; Sigma-Aldrich) and were used as mDCs. The purity of CD11c^+^ cells was greater than 90% (monoclonal antibodies against CD11c, Miltenyi Biotech, Bergisch Gladbach, Germany). Cytofluorimetric analysis was performed to evaluate the DC maturation phenotype (monoclonal antibodies against CD40, CD86, and MHC-II, Miltenyi Biotech, Bergisch Gladbach, Germany).

### DC and MSC coculture

mDCs (5 × 10^5^ cells/well) were seeded into 6-well plates 4 h after MSCs (5 × 10^6^ cells/well) in RPMI 1640 containing 5% fetal bovine serum (FBS); in the presence or absence of DAPT (5 μM) at 37 °C in 5% CO_2_. After coculture for 72 h, the suspended dendritic cells were collected for examination or applied to subsequent experiments.

### ALI model

In SPF animal room, the ALI model was induced as previously described with minor modifications [7]. Briefly, mice were intraperitoneally injected with 50 mg/kg pentobarbital. LPS (5 mg/kg) (Sigma-Aldrich) was delivered to the lungs by transtracheal injection, and the incision was sewn up. The mice were returned to the cage until they were fully awake.

### Reagent treatments

Previous studies have found that γ-secretase inhibitors impair MSC-induced DCreg production by inhibiting the Notch pathway [18, 19]. To determine the inhibitory effect of DAPT on the production of MSC-induced tolerant DCs within 3 days, MSC + DC + DAPT group: DAPT (Absin, China) was added to MSC-DC or recombinant Jagged1 cultures at 1 mΜ on day 0. When the culture medium was changed, DAPT was added to the fresh medium at the same concentrations. Because DAPT was diluted in DMSO, the control MSC-DCs, Jagged1-DCs, mDC were cultured with DMSO. GM-CSF was added to the medium in each group to maintain the final concentration of 20 ng/mL.

### Experimental groups and sample acquisition

To investigate the changes in lung DCs and pathological damage at different times after ALI modeling, the mice were randomly assigned to one of the following seven groups (n = 6 mice per group): 24 h, 12 h, 6 h, 4 h, 2 h, 1 h and 0 h. Mice in the 24, 12, 6, 4, 2, 1 and 0 h (control) groups were sacrificed after an intratracheal injection of 5 mg/kg lipopolysaccharide (LPS) (Additional file 1: Fig. S1A). Lung tissue was collected for single cell isolation and histological examination in accordance with slightly modified methods [12].

After confirming the lung DC maturation time in ALI mice, MSCs were administered to the mice at 4 h after LPS, and their effects on lung mDCs and lung pathological damage were observed. The mice were randomly assigned to one of the following three groups (n = 6 mice per group): MSC + ALI group (MSC) mice received MSCs (500,000 cells in 150 μL PBS) via the tail vein 4 h after LPS; ALI group (ALI) mice received the same amount of phosphate-buffered saline (PBS) via the tail vein 4 h after LPS; and control group (Con) mice were given the same amount of PBS at the corresponding time (Additional file 1: Fig. S1B). To confirm the effect of DCregs on CD4^+^ T cells in ALI lungs, we conducted another in vivo experiment. The control group (Con) and ALI group were treated as described above, and mice in the DCreg group (DCreg) received DCregs (500,000 cells in 150 μL PBS) via the tail vein 4 h after LPS (Additional file 1: Fig. S1C).

To confirm the effect of MSCs on DC migration in ALI mice, the following experiments were performed. ImDCs were labeled with 0.5 mM carboxyfluorescein diacetate succinimidyl ester (CFSE). A total of 1,000,000 labeled cells were injected via the tail vein into wild-type recipient mice before LPS or PBS. The mice were randomly assigned to one of the following three groups (n = 6 mice per group): MSC group (MSC) mice received MSCs (500,000 cells in 150 μl PBS) via the tail vein 4 h after LPS; ALI group (ALI) mice received the same amount of phosphate-buffered saline (PBS) via the tail vein 4 h after LPS; Control group (Con), mice were given the same amount of PBS at the corresponding time (Additional file 1: Fig. S1D).

To investigate the effect of MSC-mediated inhibition of DCreg production on the pathological damage in ALI mice, the mice were randomly assigned to one of the following two groups (n = 6 mice per group): MSC + DAPT group (MSC + DAPT) mice were injected intratracheally with DAPT (0.3 mg/kg) 0.5 h before LPS and received MSCs (500,000 cells in 150 μL PBS) via the tail vein 4 h after LPS [24]; MSC + DMSO group (MSC + DMSO) mice were injected intratracheally with the same amount of DMSO 0.5 h before LPS and received MSCs (500,000 cells in 150 μL PBS) via the tail vein 4 h after LPS; DMSO group (DMSO) mice were injected with DMSO equivalent to DAPT at 0.5 h before modeling, and were injected with the same amount of PBS with LPS or MSCs at the corresponding time; DAPT group (DAPT) mice were injected with DAPT 0.5 h before modeling, and injected with the same amount of PBS as LPS or MSCs at the corresponding time (Additional file 1: Fig. S1E). Lymphocytes in monocyte suspensions of lung tissue and peripheral blood were isolated by lymphocyte separation medium density gradient centrifugation. Lung cell isolation and measurement of the accumulation and maturation of cDCs by flow cytometry were performed as previously described [13].

### Evaluation of lung edema

Lung wet weight to body weight (LWW/BW) ratios, which reflect the severity of lung vascular permeability and lung edema, were determined for the control, ALI and MSC groups.

### Lung histopathology

The right upper lobe was embedded in paraffin and sagittally sliced into 5 μm thick sections. The sections were stained with hematoxylin and eosin. Edema, alveolar and interstitial inflammation and hemorrhage, atelectasis, necrosis, and hyaline membrane formation were each scored using a 0- to 4-point scale. The severity of lung injury was calculated as the sum of the scores as previously described [25].

### Flow cytometry

For phenotypic analysis of cell surface marker expression, cells were harvested, resuspended in PBS, incubated for 15 min with FcR blocking reagent, and then incubated for 15 min with PE-, APC-, PE-Cy7-, PerCP-, or FITC-conjugated monoclonal antibodies on ice. DCs were stained with antibodies against CD11c, CD40, CD86, CD11b, MHC-II, CD4, CD44, CD69 and PD-L1 (BD Pharmingen). Mouse IgG1 isotype control antibodies were used in parallel as negative controls. CFSE (BD Pharmingen) was used in DCs or lymphocytes. The stained cells were washed twice, resuspended in cold buffer and then analyzed by flow cytometry (FACSCalibur; NovoCyte), and the results were processed using NovoExpress software. The results are expressed as the percentage of positively stained cells relative to the total cell number.

### Mitogen proliferation assay

The protocol was based on previous mitogen proliferation and mixed lymphocyte culture with minor modifications [16]. CFSE-labeled splenocytes (5 × 10^5^ cells/well) were cocultured with allogenic DCs (mDCs or MSC-DCs, 5 × 10^4^ cells/well) in a total volume of 0.2 mL medium in 96-well U-bottom plates.

### Western blot analysis

For the Notch assay, Jagged1, Jagged2, Notch1, Notch2 and Notch3 were measured by Western blot analysis. Proteins were separated by sodium dodecyl sulfate-polyacrylamide gel electrophoresis and transferred to polyvinylidene difluoride (PVDF) membranes. The membranes were incubated with primary antibodies against Jagged1 (1:1000; Abcam), Notch3 (1:1000; Abcam), Jagged2 (1:1000; Cell Signaling), Notch1 (1:1000; Cell Signaling) and Notch2 (1:1000; Cell Signaling) at a 1:1000 dilution at 4 °C overnight. The membranes were incubated with secondary antibody for 1 h at room temperature. Immunoblots were visualized using enhanced chemiluminescence (ECL; Thermo Scientific). The expression levels from whole cell extracts were normalized to β-actin or tubulin (1:1000; Cell Signaling).

### Statistical analysis

All statistical analyses were performed using SPSS 23.0 software and GraphPad Prism 7.0. One-way analysis of variance (ANOVA) or two-tailed Student’s t test was used to determine the significance between the groups. The data are expressed as the mean ± standard deviation (SD). A value of p < 0.05 was considered significant.

## Results

### Lung cDCs increase in ALI mice

**Fig. 1:**
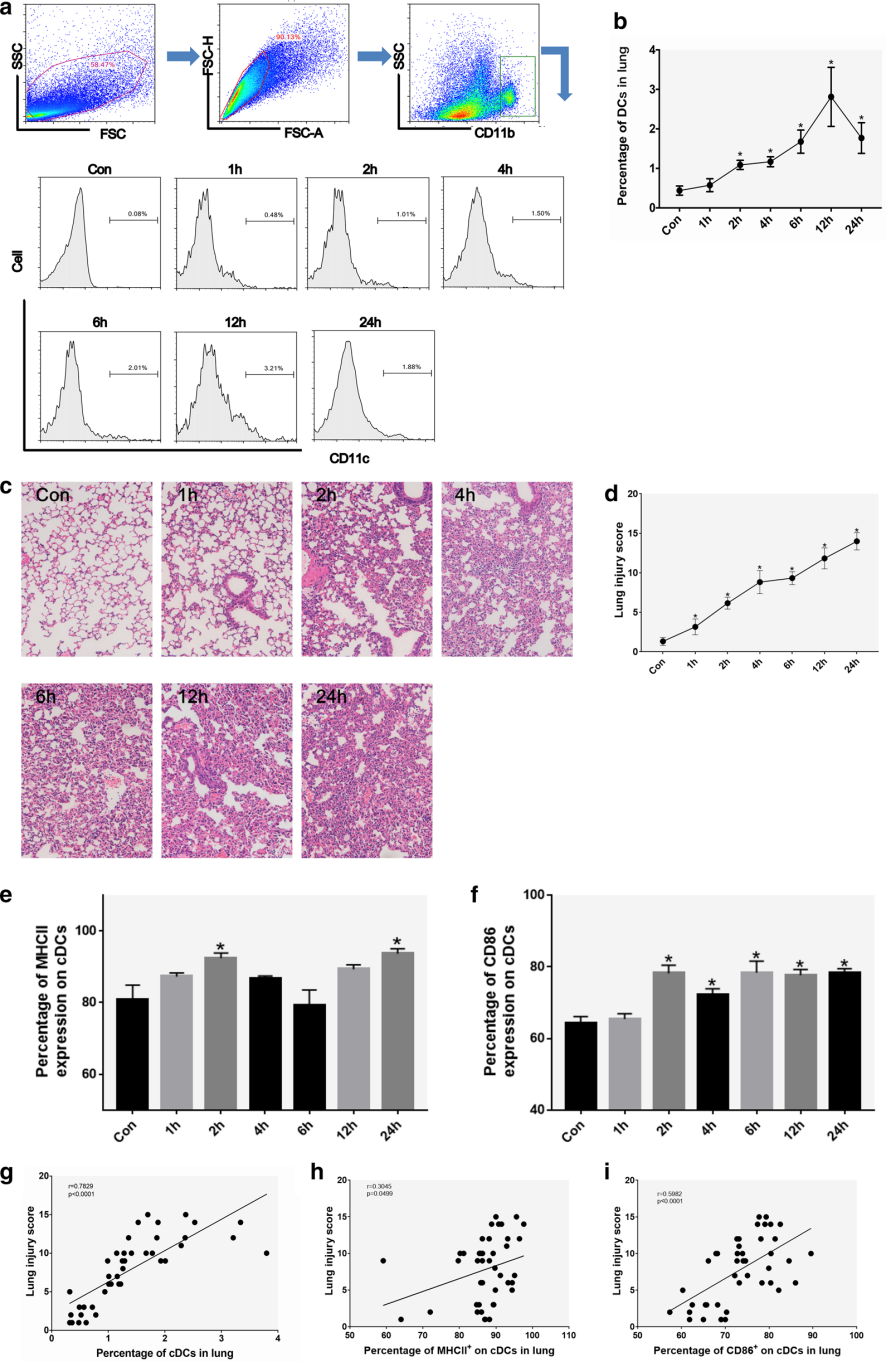
The percentage of cDCs and mDCs in the lungs of ALI mice and the degree of pathological damage at different times after intratracheal injection of LPS. **a**, **b** The percentage of cDCs changed with time after intratracheal LPS injection in ALI mice. **c**, **d** Representative tissue sections of lung tissue (hematoxylin and eosin staining; magnification, ×200) and pathological lung injury scores from 0 to 24 h. **e**, **f** Expression of MHCII and CD86 in cDCs in the mouse lung cells from 0 to 24 h after intratracheal LPS injection. **g**–**i** The correlation between the percentage of cDCs, MHCII^+^ cDCs and CD86^+^ cDCs and pathological scores was analyzed by Pearson correlation coefficient. The data are presented as the mean ± SD. *p < 0.05 vs. the control group; n = 6 per group

We first investigated the percentage of CD11c^+^ cDCs in lung tissue in LPS-induced mice by flow cytometry. The percentage of cDCs in ALI mice gradually increased over time after LPS intratracheal injection (especially after 2 h) compared with that of the controls (Con) but decreased after 12 h (Fig. 1a, b), and the degree of pathological damage in the lungs gradually worsened (Fig. 1c), including hemorrhage, increased edema, inflammatory cell infiltration, and increased atelectasis, resulting in a progressively increased lung injury score (Fig. 1d). In addition, ALI mice showed a gradual increase in the percentage of mDCs in lung tissue (especially CD86^+^ DCs) after intratracheal injection of LPS (Fig. 1e, f). The percentage of cDCs and mDCs was positively correlated with the ALI lung pathological injury score (Fig. 1g–i). These results indicate that cDCs, especially mDCs, participate in the progression of ALI pathogenesis.

### MSCs inhibit the recruitment and maturation of cDCs in ALI mice and alleviate lung pathological damage

**Fig. 2:**
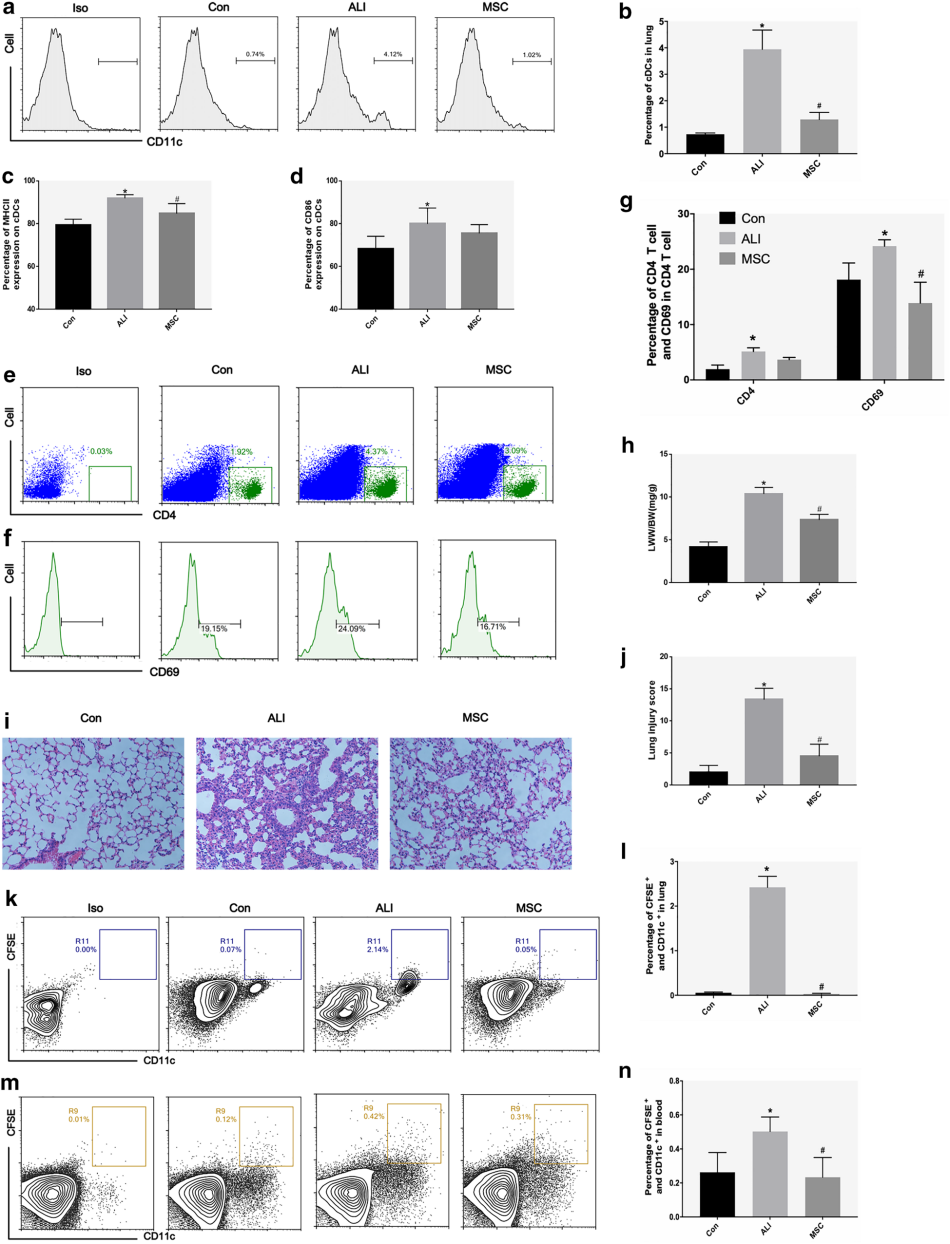
Tail vein injection of MSCs inhibited the recruitment and maturation of lung cDCs in ALI mice and improved pulmonary damage. **a**, **b** The percentage of lung cDCs in normal, ALI and MSC groups. **c**, **d** The percentage of MHCII^+^ or CD86^+^ cells in lung cDCs of mice in normal, ALI and MSC groups. **e**–**g** The percentages of lung CD4^+^ T cells and CD69^+^ cells in CD4^+^ T cells in the normal, ALI, and MSC groups. **h** Lung wet weight to body weight ratio (LWW/BW) of mice in normal group, ALI group and MSC group. **i**, **j** Histopathological features of lung (hematoxylin and eosin staining; magnification, ×200) and lung injury score in the normal, ALI, and MSC groups. **k**, **l** The percentage of CFSE-labeled DCs in peripheral blood in normal group, ALI group and MSC group. **m**, **n** The percentage of CFSE-labeled DCs in the lung of mice in normal group, ALI group and MSC group. The data are presented as the mean ± SD. *p < 0.05 vs. the control group, ^#^p < 0.05 vs. ALI group; n = 6 per group

In view of the significant changes in lung DCs 2 h after airway injection of LPS, the mice were treated with MSCs 4 h after LPS administration. The percentage of cDCs was significantly decreased compared with that of ALI mice 24 h after MSC treatment (Fig. 2a, b). The proportion of mDCs (MHCII^+^ DCs) among the lung cDCs of ALI mice was also significantly reduced after MSC treatment (Fig. 2c, d). The percentages of CD4^+^ T cells and CD69^+^ in CD4^+^ T cells in the lung were significantly increased in the ALI group and decreased after treatment with MSC (Fig. 2e–g). In addition, after MSC treatment, lung pathological injuries, such as hemorrhage, edema, inflammatory cell infiltration, and atelectasis, were all significantly reduced (Fig. 2i), and the pathological scores of the lung and LWW/BW were also decreased (Fig. 2h, j). Our findings suggest that MSCs reduce ALI lung injury, accompanied by a decrease in the number and maturity of cDCs in the lungs. The in vivo results showed that CFSE-labeled DCs increased in the blood and lung tissues of ALI mice compared with the control group (Fig. 2k, l). After MSC treatment, the number of CFSE-labeled DCs in the blood and lung tissues of ALI mice decreased (Fig. 2m, n).

### MSCs induce the production of immunotolerant DCs

**Fig. 3:**
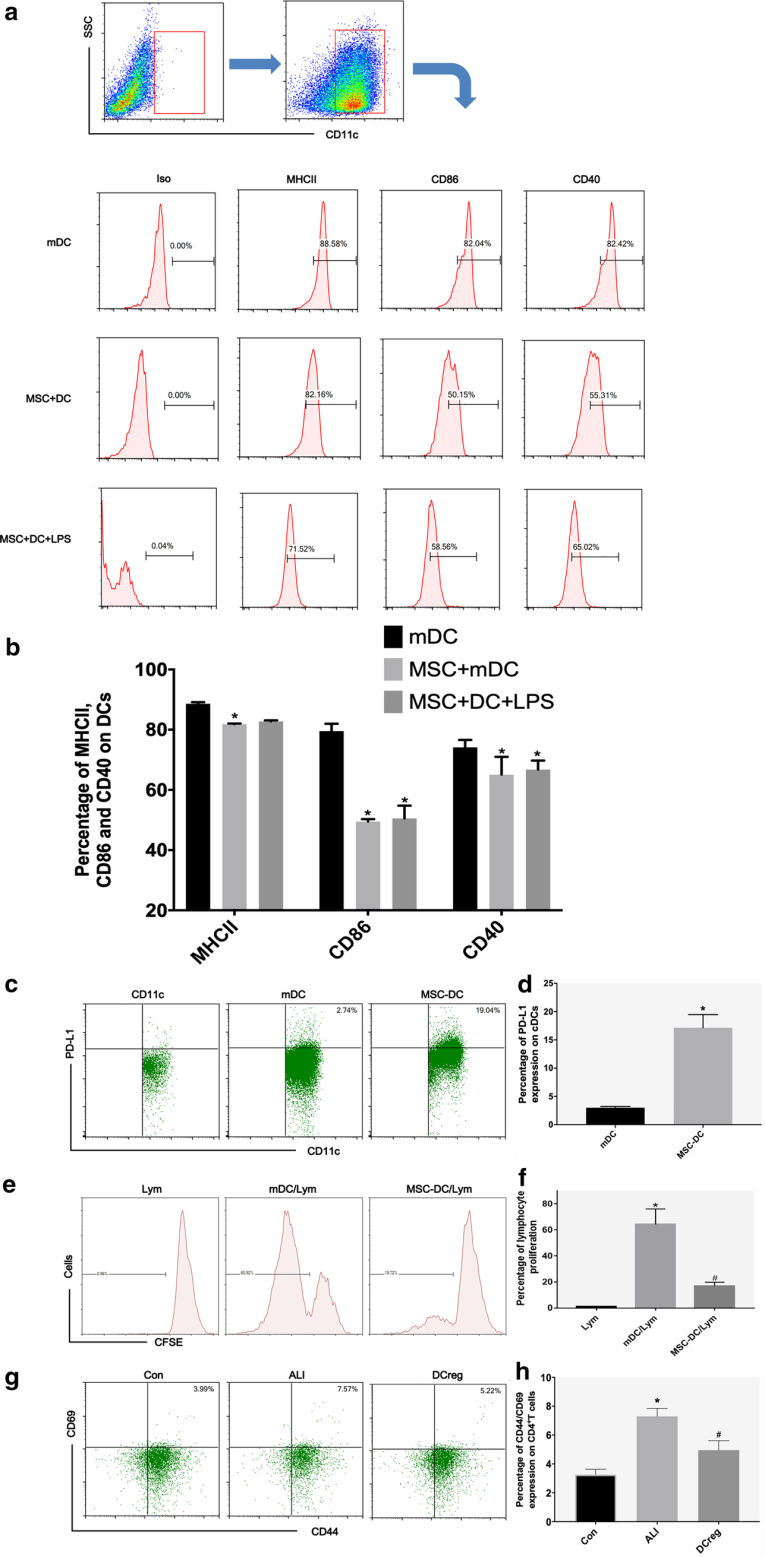
MSCs induced the production of immunotolerant DCs by cell–cell interactions. **a**, **b** The percentages of MHCII^+^, CD86^+^ and CD40^+^ based on CD11c^+^DC gate in mDC, MSC + mDC and MSC + mDC + LPS groups. **c**, **d** The percentages of PD-L1 + DCs based on CD11c + DC gate in mDC, MSC + mDC and MSC + mDC + LPS groups. **e**, **f** The percentage of lymphocyte proliferation in mDC group, MSC + mDC group and MSC + mDC + LPS group. **g**, **h** The percentages of CD44^+^ CD69^+^ based on CD4^+^ T cell gate in mDC, MSC + mDC and MSC + mDC + LPS groups. n = 3 per group; the data are presented as the mean ± SD. **b**, **d** *p < 0.05 vs. the mDC group, ^#^p < 0.05 vs. MSC + mDC group; **f** *p < 0.05 vs. the Lym group, ^#^p < 0.05 vs. the mDC/lym group; **h** *p < 0.05 vs. the Con group, ^#^p < 0.05 vs. the ALI group

The in vivo experiments indicated that the cDCs in the lung were mainly mDCs as early as 2 h after intratracheal injection of LPS, and MSC treatment reduced the maturity of DCs. Therefore, we further investigated whether MSCs affect the phenotype and function of mDCs through direct contact by in vitro coculture and found that DCs cocultured with MSCs expressed decreased levels of MHCII, CD86, and CD40 compared to those of mDCs, and the effect of MSC on mDCs was not changed even in the LPS environment (Fig. 3a, b). By examining the protein expression of the immunotolerance marker PD-1, it was found that MSCs reduced the expression of PD-1 in mDCs (Fig. 3c, d). A mitogen proliferation assay was performed to verify functional changes in MSC-DCs. CFSE-labeled splenic lymphocytes were used as responders and were cocultured with mDCs and MSC-DCs. The results showed that the ability of MSC-DCs to stimulate lymphocyte proliferation was significantly weaker than that of mDCs (Fig. 3e, f). These results demonstrate that MSCs induce mDCs into immune-tolerant DCs that inhibit lymphocyte proliferation. In addition, we further evaluated the effect of MSC-induced DCregs on the activation of pulmonary CD4^+^ T cells in LPS-ALI mice. The results showed that the expression of CD44^+^CD69^+^ in pulmonary CD4^+^ T cells was increased in ALI mice but decreased significantly after DCreg treatment (Fig. 3g, i).

### The Notch pathway is activated during MSC-induced differentiation of mDCs into DCregs

**Fig. 4:**
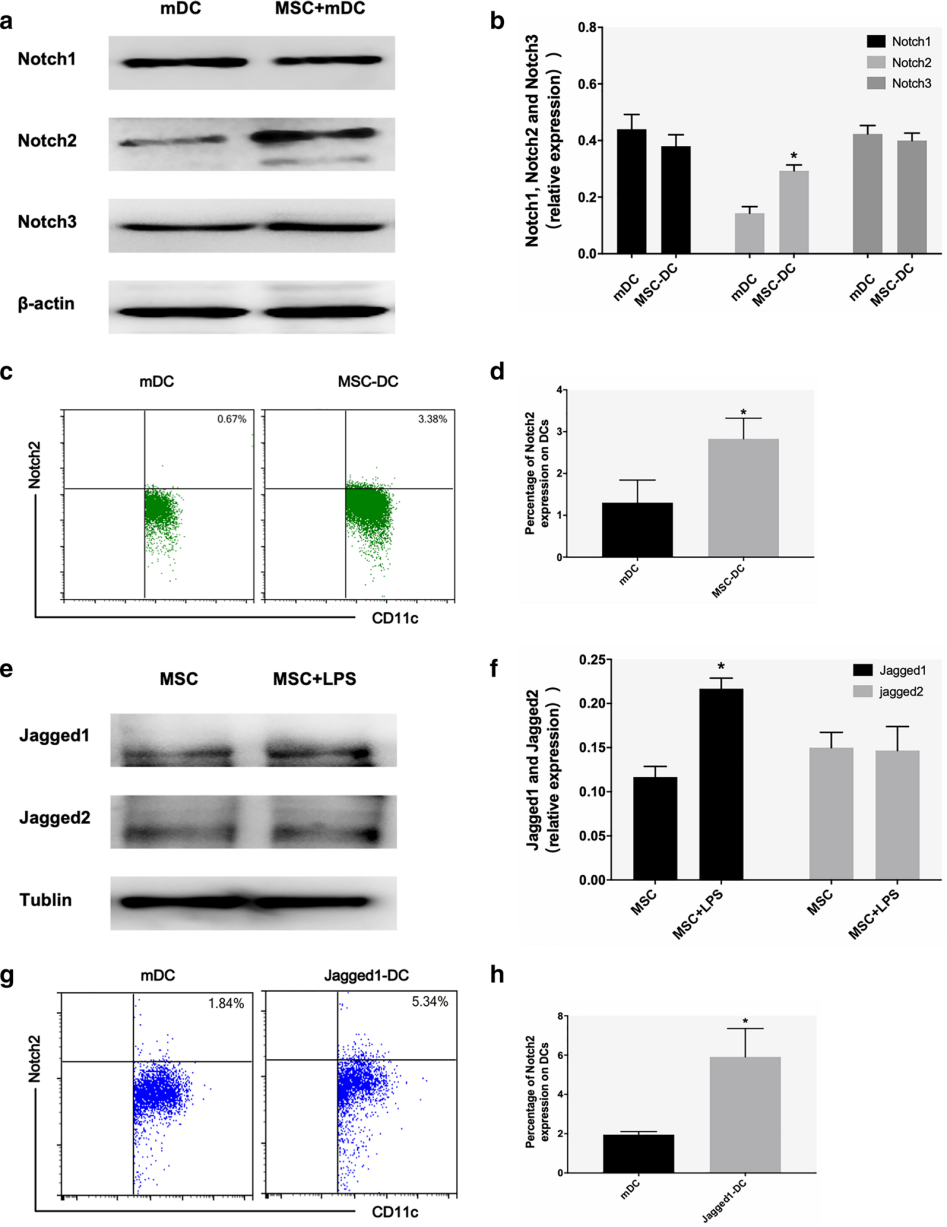
The Notch pathway is activated during the MSC-induced differentiation of mDCs into DCregs. **a**, **b** The expression of Notch1, Notch2 and Notch3 protein levels in DCs that were cultured with or without MSCs for 72 h was evaluated using Western blot analysis. **c**, **d** The percentage of Notch2-positive cells in CD11c^+^ DCs that were cultured with or without MSCs for 72 h was detected by flow cytometry. **e**, **f** The expression of Jagged1 and Jagged2 protein levels in MSCs that were cultured with or without LPS for 24 h was evaluated using Western blot analysis. **g**, **h** The percentage of Notch2-positive cells in CD11c^+^ DCs that were cultured with or without rhjagged1 for 72 h was detected by flow cytometry. n = 3, **b**, **d** and **h** *p < 0.05 versus mDC; **f** *p < 0.05 versus MSC; the data are expressed as the mean ± SD

The Notch pathway plays an important role in regulating dendritic cell differentiation through direct cell-to-cell contact mechanisms [21]. Previous studies have found that the Notch pathway is activated during MSC-induced tolerance of DCs to inflammation in mice with allergic airway inflammation. To confirm the involvement of the Notch pathway in the induction of DCregs by MSCs, we detected DC Notch receptor protein expression after 3 days of coculture with MSCs. We measured the protein levels of Notch1, 2 and 3 in DCs after coculture by Western blotting. Surprisingly, we found that mDCs that were cocultured with MSCs contained more Notch2 than mDCs (Fig. 4a, b). The flow cytometry results also showed that the expression of Notch2 in CD11c^+^ cells was higher than that in DCs after coculture (Fig. 4c, d). Similarly, we also detected the expression of jagged1 and 2 in MSCs, and MSCs expressed more jagged1 protein under LPS stimulation (Fig. 4e, f). The percentage of Notch2^+^DC after rhJagged1 treatment was significantly higher than that of mDC (Fig. 4g, h). Collectively, these data show that the Notch pathway is activated during MSC-induced differentiation of mDCs into DCregs, which may be an important mechanism by which MSCs regulate DC function.

### MSCs induce the differentiation of mDCs into DCregs via the Notch signaling pathway

**Fig. 5:**
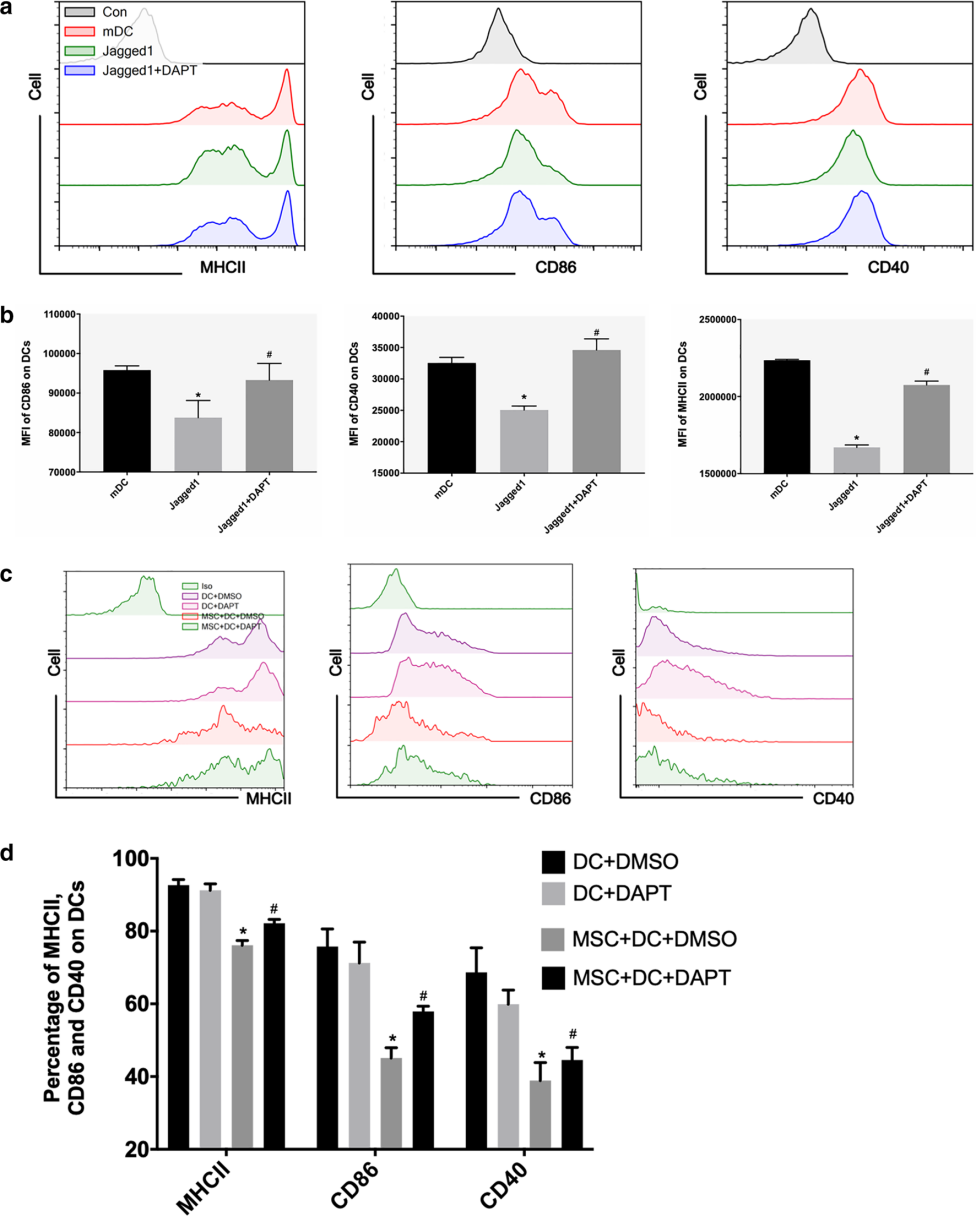
MSCs induce the differentiation of mDCs into DCregs via the Notch signaling pathway. **a**, **b** The percentages of MHCII^+^, CD86^+^ and CD40^+^ based on CD11c^+^DC gate in mDC, Jagged1 and Jagged1+ DAPT groups. **c**, **d** The percentages of MHCII^+^, CD86^+^ and CD40^+^ based on CD11c^+^DC gate in DC + DMSO, DC + DAPT, MSC + DC + DMSO and MSC + DC + DAPT groups. n = 3, **b** *p < 0.05 versus mDC, ^#^p < 0.05 versus Jagged1; **d** *p < 0.05 versus MSC + DC + DMSO; the data are expressed as the mean ± SD

The Notch pathway plays an important role in regulating dendritic cell differentiation through direct cell-to-cell contact mechanisms. Therefore, we first verified whether recombinant mouse Jagged1 induced mDC differentiation into immune-tolerant DCs. Jagged1 decreased the expression of DC MHCII, CD86 and CD40, but this effect was offset by a gamma secretase inhibitor (DAPT) (Fig. 5a, b). Subsequently, we determined the DC phenotype induced by MSCs in the presence or absence of a gamma secretase inhibitor (DAPT) by flow cytometry in vitro. In contrast to the results shown in Fig. 3a, b, DAPT increased the expression of MHCII and CD86 in DCs that were cocultured with MSCs (Fig. 5c, d). These data suggest that inhibition of the Notch pathway impairs MSC-induced DCreg production, but the in vivo results are still unclear.

### MSCs induced regulatory DCs to attenuate ALI via the notch pathway

**Fig. 6:**
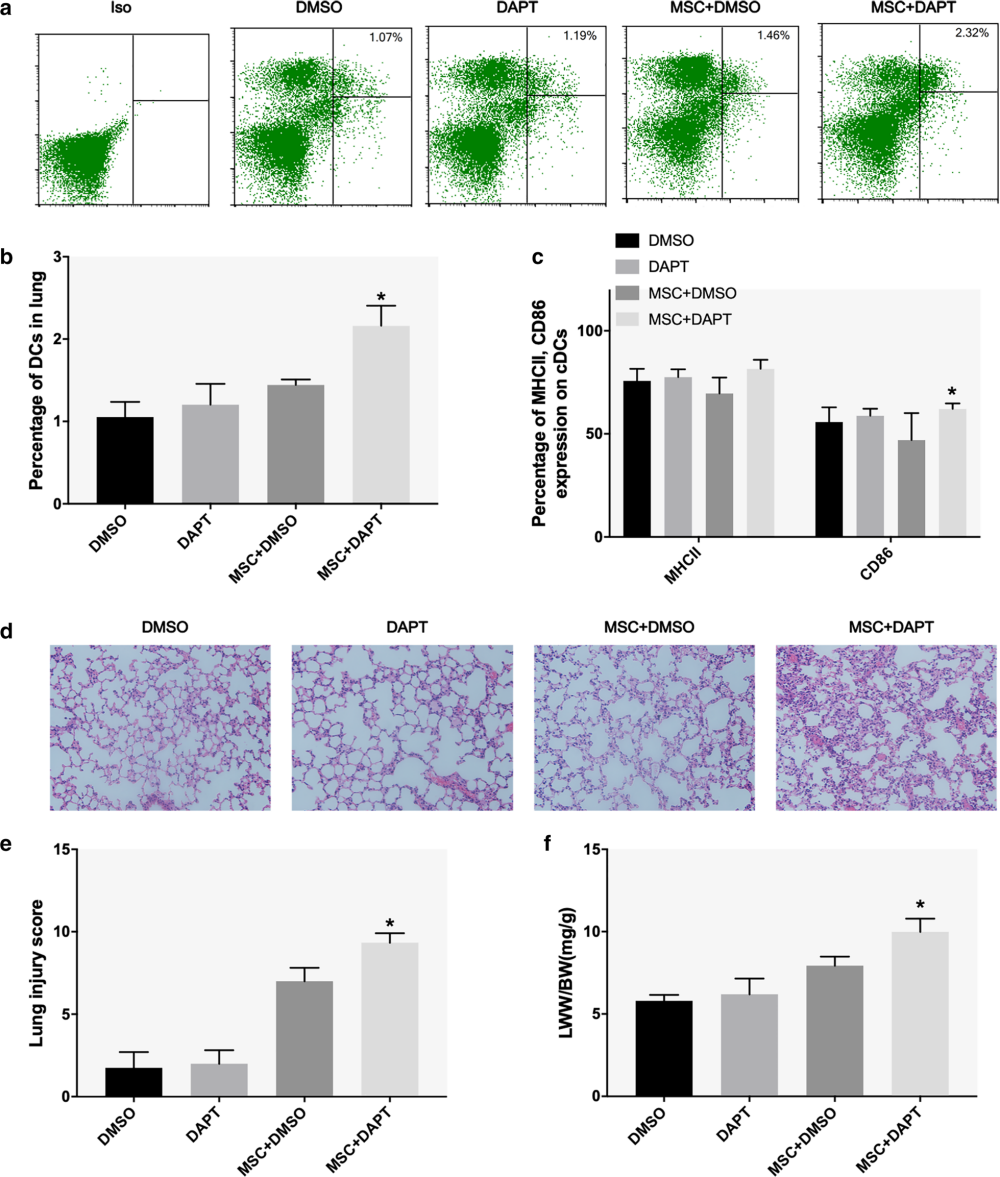
MSCs alleviate acute lung injury by inducing tolerant DCs. **a**, **b** The percentage of lung cDCs (CD11c^+^CD11b^+^ DCs) in the DMSO, DAPT, MSC + DMSO, and MSC + DAPT groups. **c** The expression of MHCII and CD86 in lung cDCs in the DMSO, DAPT, MSC + DMSO, and MSC + DAPT groups. **d**, **e** Histopathological features of lung (hematoxylin and eosin staining; magnification, ×200) and lung injury score in the DMSO, DAPT, MSC + DMSO, and MSC + DAPT groups. **f** The ratio of lung wet weight to body weight (LWW/BW) in the DMSO, DAPT, MSC + DMSO, and MSC + DAPT groups. The data are presented as the mean ± SD. *p < 0.05 vs. the MSC + DMSO group

Figure 2 shows that MSC treatment alleviated lung injury and reduced the number and maturity of respiratory cDCs. DCreg generation induced by MSCs was inhibited by DAPT in vitro. To further investigate the role of the Notch pathway in MSC-mediated improvement in lung injury and lung DCs in vivo, the inhibitor DAPT was used. We found that after DAPT intervention, the inhibitory effect of MSCs on CD11c^+^11b^+^ DC recruitment among lung cells was weakened (Fig. 6a, b). Similarly, DAPT inhibition of the NOTCH pathway reversed the effect of MSCs on the lung DC phenotype and increased the antigen-presenting molecule MHCII and costimulatory molecule CD86 (Fig. 6c). In addition, after DAPT treatment, lung tissue pathological injury was significantly aggravated, presenting as increased pulmonary hemorrhage, edema, inflammatory cell infiltration and atelectasis (Fig. 6d). Lung injury score and the LWW/BW were increased (Fig. 6e, f).

## Discussion

In this study, we found significant accumulation and maturation of lung DCs 2 h after intratracheal injection of LPS, which were positively correlated with the lung pathological injury score. MSCs induce mDCs to differentiate into DCregs, which have low expression of MHCII, CD86 and CD40, by direct cell–cell contact. In addition, we also showed that MSCs activate the Notch pathway by Jagged1 binding and increasing the expression of Notch2 to induce the generation of DCregs. On the other hand, by inhibiting the Notch pathway in vivo and in vitro, the induction of DCregs by MSCs was weakened, and the pulmonary protective effect of MSCs on ALI was reduced. In conclusion, MSCs induce DCreg production by activating the Notch pathway to alleviate acute lung injury.

Previous studies showed that mature DCs increased significantly in the lung tissues of ALI mice, and Fms-like tyrosine kinase 3 pretreatment stimulates lung DC proliferation and maturation, exacerbating lung pathological damage. In contrast, Losartan pretreatment inhibited lung DC proliferation and maturation and alleviated lung damage [12, 13]. These results indicated that mature DCs play a key role in the pathogenesis of ALI. We found that cDCs in the lung tissues of ALI mice matured and increased significantly as early as 2 h after LPS injection and continued to increase up to 12 h. Although we found a positive correlation between the percentage and maturity of cDCs and the lung pathological injury score, the contribution of intervention with mature DC function to lung protection in ALI should be interpreted cautiously. On the one hand, the evidence is that LPS can stimulate the mature activation of DC, and mature DCs express a large number of MHC II and CD80, CD86 and other co-stimulating molecules, inducing the activation of naive T cells and differentiating into effector T cells [26], which aggravate the inflammatory response of ALI [27]. Therefore, regulating the functions of mature DCs may be crucial in LPS-induced ALI treatment. On the other hand, respiratory DCs sample microorganisms and particulates from the respiratory tract and present them to T cells to initiate an adaptive immune response to clear bacteria in the lungs [28]. In fact, the regulation of DC therapy for bacteria-induced ARDS requires finding a balance between the elimination of pathogens and the suppression of organ damage caused by excessive inflammatory responses. However, in this study, we observed the effect and mechanism of MSC on lung DCs in LPS-induced-ALI mice without considering the factor of bacteria.

MSCs show strong immunoregulatory functions by suppressing T cell proliferation and activation, inducing regulatory T cells, inhibiting B cell function, or promoting the phagocytic activity of macrophages [8–10]. However, whether DCs are regulated by MSCs in the ALI microenvironment is not known. Our findings revealed that MSCs significantly downregulated the percentage and maturity of cDCs in ALI mice and alleviated lung injury. According to the findings of this study, the percentage of mDCs increased significantly in ALI mice 2 h after LPS administration but decreased after treatment with MSCs, suggesting that MSCs induce cDCs from mature phenotypes to differentiate into immature phenotypes, alleviating lung injury and reducing pulmonary edema. To support this conclusion, we performed in vitro experiments that showed that mDC expression of PD-L1, the antigen presenting molecule MHCII and costimulatory molecules CD86 and CD40 decreased significantly after 3 days of coculture with MSCs, and still maintained stable phenotypes in the LPS microenvironment, which was consistent with the previous 7 days of coculture [16]. The expression levels of MHCII, CD86 and CD40 determine whether DCs can capture and process antigens and deliver them to T cells, which affects the proliferation and activation of T cells [29, 30], and PD-1 is necessary for DC-mediated induction of regulatory T cells and tolerance [31]. We confirmed that the proliferation and activation of T cells that were stimulated by DCs after coculture with MSCs were decreased, and we also found that treatment with MSC reduced the number and percentage activation of CD4^+^T cells in the lung of ALI mice.

Studies have reported that after MSC culture with DCs, the expression of CCR7 was decreased after DC stimulation, and migration to CCL19 or CCL21 was also significantly reduced [15, 32]. Analysis of respiratory dendritic cell subsets revealed significantly reduced lung DC infiltration in MSC-treated mice with acute lung injury induced by *Klebsiella pneumoniae* [33], and Chiesa also reported that MSCs inhibit DC migration to lymph nodes [34]. Consistent with these results, we found that lung DCs were significantly reduced in ALI mice that were treated with MSCs, which may be due to MSC-mediated inhibition of DC migration. The results of in vivo experiments showed that CFSE-labeled DCs had increased retention times in ALI mouse blood, indicating that MSCs reduced the retention of CFSE-labeled DCs in ALI mouse blood, resulting in reduced migration of DCs to the lungs.

The Notch signaling pathway controls cell proliferation, apoptosis, survival and differentiation during cell development and homeostasis [21, 35–38]. MSCs induced a semimature DC phenotype that required jagged1 to activate Notch signaling for the expansion of regulatory T cells, reducing the pathology in a mouse model of allergic airway inflammation [19]. Consistent with these results, our study shows that under LPS stimulation, MSCs expressed more jagged1, and both MSCs and recombinant jagged1 induced the generation of DCregs. Jagged1/Notch2 signal activation is closely related to cell regeneration and immune cell regulation [39, 40]. Previous studies have shown that promoting the expression of NOTCH2 reduces the efficiency of DC presentation of MHC class II-restricted antigens and limits the strength of CD4^+^ T cell activation [41]. This study similarly found that the expression of Notch2 receptor protein was significantly increased in MSC-treated DCs or recombinant jagged1-treated DCs. Therefore, these results suggest that the Notch pathway is involved in the mechanism by which MSCs induce mDC immune tolerance. In this study, the expression of costimulatory molecules in DCs and functional markers of T cells that were stimulated by DCs showed that MSCs induced DCreg production.

γ-Secretase inhibitors are a class of small molecular compounds that target the Notch pathway and have been used in preclinical and clinical trials to treat a variety of diseases [42, 43]. Gamma-secretase inhibitors have been shown to attenuate neurogenic acute lung injury in rats [44]. This study found that MSCs reduced the expression of DC costimulatory molecules and functional markers, which were blocked by the γ-secretase inhibitor DAPT. Therefore, we performed in vivo experiments and confirmed that induction of tolerant DCs is a key link in MSC-mediated alleviation of lung pathological damage by inhibiting the Notch pathway. These data suggest that MSCs induce tolerant DCs by activating the Notch pathway to alleviate acute lung injury.

The LPS-induced ALI model has some limitations compared with the bacteria-induced animal model. LPS is a potent agent that activates the innate immune response via the TLR4 pathway, and its use provides information about the effects of host inflammatory response, which occurs in bacterial infections [45]. Many previous studies selected LPS-induced ALI model to study the function of immune cells in ARDS [46, 47]. In addition to endotoxins, gram-negative bacteria (*E. coli*) also continue to produce a variety of virulence factors such as adhesin, exotoxin and type III secretion system. The adhesin produced by bacteria mediates the adhesion between bacteria and epithelial cells [48]. Bacteria produce potent cytotoxic exotoxins [49] as well as utilize the type III secreting system to directly cause epithelial cell lysis [50]. This is consistent with Wiener-Kronish’s findings that LPS treatment does not cause the severe endothelial injury that occurs in ARDS [51]. LPS binds to specific LPS binding proteins to form a complex, which activates the CD14/TLR4 receptor structure mostly on the surface of immune cells, such as monocytes, macrophages, and dendritic cells, triggering the production of inflammatory mediators [52]. This study focused on the effect of MSC treatment on DC function in ALI, so LPS-induced animal model was selected. In addition, LPS is easy to administer and has little direct toxicity to cultured cells in vitro [45].

## Conclusions

This study demonstrated that the recruitment and maturation of lung DCs is an important process in early acute lung injury. The induction of tolerant dendritic cells is a crucial part of MSC-mediated alleviation of early acute lung injury, and this process is closely related to activation of the Notch pathway. These findings provide new insights into the role of MSCs in lung injury repair.

## Supplementary information

**Additional file 1: Fig. S1.** Time schedule of drug or cell injections in vivo. (A) Changes of lung DC at different time after intratracheal injection of LPS: time schedule for drug or cell injection. (B) Effect of MSC therapy on lung DCs in ALI mice: time schedule for drug or cell injection. (C) Effects of DCreg therapy on the activation of CD4^+^T cells in the lungs of ALI mice: time schedule for drug or cell injection. (D) Effect of MSCs on DC migration from peripheral blood to lung in ALI mice: time schedule for drug or cell injection. (E) Effect of DAPT on the regulation of lung DC function by MSC in ALI mice:time schedule for drug or cell injection.

## Data Availability

Not applicable.

## Abbreviations

ARDS: Acute respiratory distress syndrome
DCs: Dendritic cells
ALI: Acute lung injury
cDCs: Conventional DCs
DCregs: Regulatory DCs
imDCs: Immature DCs
mDCs: Mature DCs
MSCs: Mesenchymal stem cells
LPS: Lipopolysaccharide
PD-L1: Programmed death ligand 1
BM: Bone marrow
FBS: Fetal bovine serum
GM-CSF: Granulocyte-macrophage colony- stimulating factor
IL-4: Interleukin-4
LWW/BW: Lung wet weight to body weight ratio
CFSE: Carboxyfluorescein diacetate succinimidyl ester
PVDF: Polyvinylidene difluoride
PBS: Phosphate-buffered saline
ANOVA: Analysis of variance
SD: Standard deviation
