# Supplementary figures and images for: Mesenchymal stem cells activate Notch signaling to induce regulatory dendritic cells in LPS-induced acute lung injury
Source: J Transl Med. 2020 Jun 16;18:241. doi: 10.1186/s12967-020-02410-z (PMC7298963; doi:10.1186/s12967-020-02410-z)

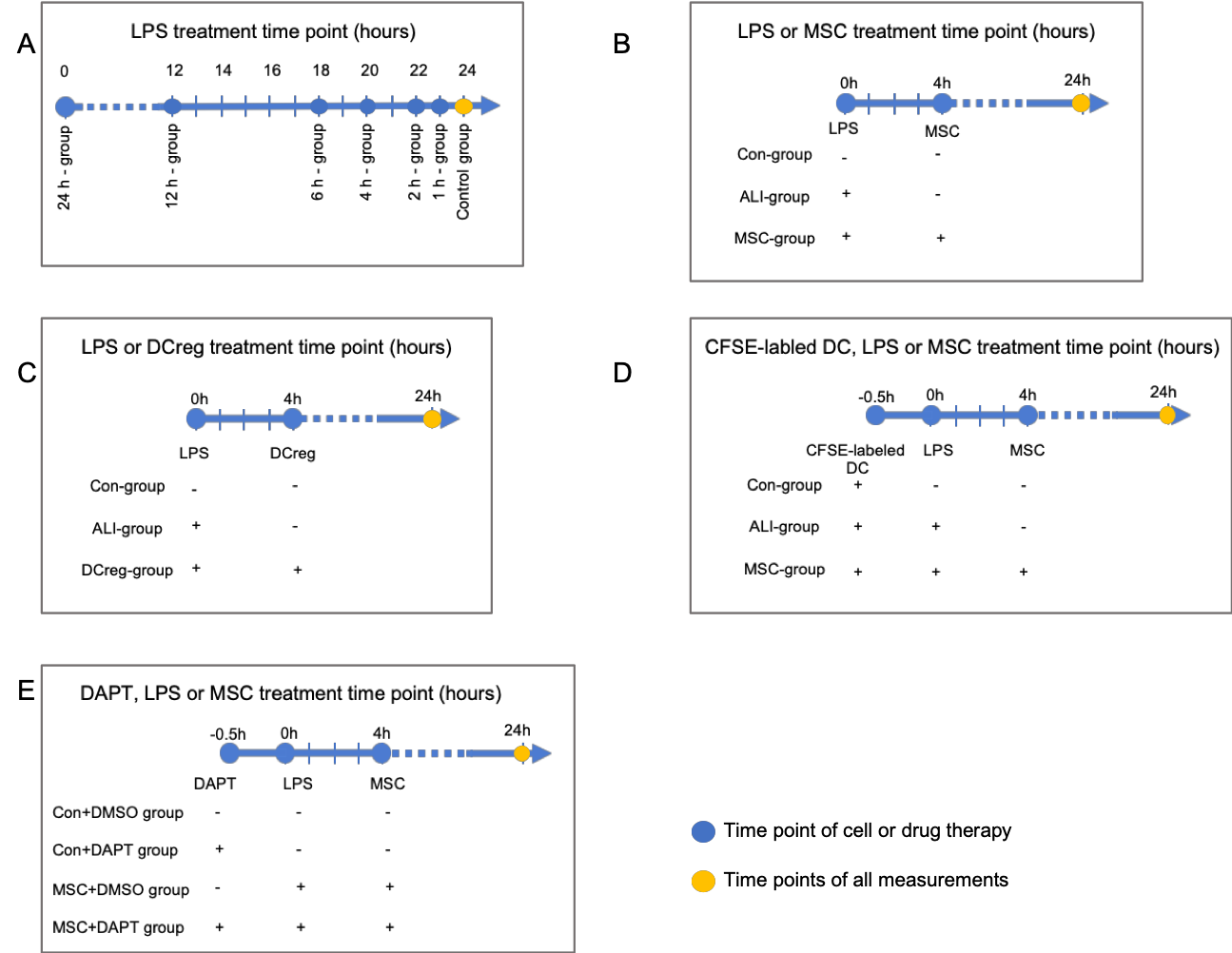

Supplement: Supplementary file 1 — Additional file 1: Fig. S1. Time schedule of drug or cell injections in vivo. (A) Changes of lung DC at different time after intratracheal injection of LPS: time schedule for drug or cell injection. (B) Effect of MSC therapy on lung DCs in ALI mice: time schedule for drug or cell injection. (C) Effects of DCreg therapy on the activation of CD4+T cells in the lungs of ALI mice: time schedule for drug or cell injection. (D) Effect of MSCs on DC migration from peripheral blood to lung in ALI mice: time schedule for drug or cell injection. (E) Effect of DAPT on the regulation of lung DC function by MSC in ALI mice:time schedule for drug or cell injection. [file 12967_2020_2410_MOESM1_ESM.tif]
